# The diversity and abundance of fungi and bacteria on the healthy and dandruff affected human scalp

**DOI:** 10.1371/journal.pone.0225796

**Published:** 2019-12-18

**Authors:** Sally G. Grimshaw, Adrian M. Smith, David S. Arnold, Elaine Xu, Michael Hoptroff, Barry Murphy

**Affiliations:** 1 Unilever Research & Development, Port Sunlight, England, United Kingdom; 2 Unilever Research & Development, Colworth, England, United Kingdom; 3 Unilever Research & Development, Shanghai, China; Skin Research Institute Singapore, SINGAPORE

## Abstract

Dandruff is a skin condition that affects the scalp of up to half the world’s population, it is characterised by an itchy, flaky scalp and is associated with colonisation of the skin by *Malassezia* spp. Management of this condition is typically via antifungal therapies, however the precise role of microbes in the aggravation of the condition are incompletely characterised. Here, a combination of 454 sequencing and qPCR techniques were used to compare the scalp microbiota of dandruff and non-dandruff affected Chinese subjects. Based on 454 sequencing of the scalp microbiome, the two most abundant bacterial genera found on the scalp surface were *Cutibacterium* (formerly *Propionibacterium*) and *Staphylococcus*, while *Malassezia* was the main fungal inhabitant. Quantitative PCR (qPCR) analysis of four scalp taxa (*M*. *restricta*, *M*. *globosa*, *C*. *acnes* and *Staphylococcus* spp.) believed to represent the bulk of the overall population was additionally carried out. Metataxonomic and qPCR analyses were performed on healthy and lesional buffer scrub samples to facilitate assessment of whether the scalp condition is associated with differential microbial communities on the sampled skin. Dandruff was associated with greater frequencies of *M*. *restricta* and *Staphylococcus* spp. compared with the healthy population (p<0.05). Analysis also revealed the presence of an unclassified fungal taxon that could represent a novel *Malassezia* species.

## Introduction

It is well accepted that the human body’s resident microbiota play a critical role in the maintenance of human health with numerous studies demonstrating that alterations in microbial community composition are associated with disease states as diverse as irritable bowel syndrome, acne, periodontal diseases and atopic dermatitis [1–3]. The proposed microbiome-mediated cosmetic condition dandruff is a scalp condition characterised by excessive flaking of skin, often accompanied by dryness and itch [4].

Over half of the world’s population will suffer from dandruff during their lifetime [5]. Despite its prevalence the aetiology of dandruff has been the subject of much debate [6]. A general consensus has emerged: increased colonisation by yeasts of the genus *Malassezia* [7]; some element of host predisposition [8]; sebaceous gland activity [8]; deteriorated integrity of the scalp stratum corneum [9, 10] and a host inflammatory response. Each potentially play a role in the pathobiology of dandruff.

The genus *Malassezia* comprises a group of lipophilic yeasts which lack the metabolism necessary for fatty acid synthesis [11]. In total 18 members of the genus [12–15] are described, most of these have been examined in-depth via genus wide comparative genomics by Wu *et al* [12].

The association between altered relative and absolute abundance of *Malassezia* spp. and scalp health was shown in multiple studies [16–18]. These fungi are considered commensal members of the human skin microbiome [19], where they are predominately represented by *M*. *restricta* and *M*. *globosa*. These and other microbial species were found to be associated with dandruff and other human skin conditions [20, 21]. A causative link was made due to the observed reduction in *Malassezia* numbers, and subsequent reduction in scalp skin flaking, following the application of a shampoo containing antifungal compounds such as zinc pyrithione, ketoconazole, piroctone olamine and climbazole [22–24]. In addition, the cessation of treatment in some individuals results in an increased level of skin flaking [25]. However, an exacting causative relationship between *Malassezia* spp. and dandruff is confounded by the high prevalence of *Malassezia* on both healthy and dandruff affected skin [26, 27]. This has led to the development of a susceptibility hypothesis where both altered levels of *Malassezia* spp. as well as host predisposition are required for disease progression [28]. While a definitive mechanism has yet to be established, it is considered likely that an underlying skin barrier defect comprising increased keratinocyte turnover, decreased epidermal thickness and reduced stratum corneum lipid content are important in the onset of dandruff symptoms. These potential predisposing factors may facilitate the adsorption of potentially irritant unsaturated fatty acids and other metabolites produced by *Malassezia* contributing to disease progression [4, 9, 29].

Seasonality may also play a contributory environmental role, with the dandruff condition being exacerbated during the winter months [4]. Hypotheses for a seasonal effect range from a decrease in stratum corneum lipid levels [30] to changes in the levels of sebum on skin [31, 32] and facial scaling [33].

While historically less associated with dandruff, the scalp bacterial microbiome has recently become the focus of some attention for a potential causative role in dandruff progression [34]. Analysis has shown that in both French and Chinese populations that *Staphylococcus* and *Cutibacterium* (formerly *Propionibacterium*) dominate healthy and dandruff scalps [16, 17]. Both studies were carried out using cloning approaches as opposed to next generation sequencing (NGS) however in both instances the ratio of *Staphylococcus* to *Cutibacterium* increased on dandruff scalps. These findings were confirmed in an NGS based study where Xu *et al*., showed that the association between bacterial frequencies and dandruff was stronger than the association between fungal frequencies and dandruff [34].

Recent developments in next generation sequencing have facilitated the high throughput analysis of microbial communities at a level not possible only a few years ago. The work here outlines a metataxonomic profiling approach taken to determine the scalp microbial profile of the resident bacterial and fungal communities on both healthy and dandruff scalps. Relative abundance assessment provided by metataxonomic analysis is supplemented by qPCR of the key bacterial and fungal community members of the scalp microbiome. Our results show that characteristic bacterial and fungal communities are associated with dandruff and non-dandruff scalps, while adding quantitative assessment of targeted Staphylococcus species not previously described.

## Materials and methods

### Study design

Chinese subjects aged between 18 and 65 years were recruited through the Unilever Shanghai Clinical Research Centre, China. Written informed consent was obtained from all participants. The study was reviewed and approved by the Independent Ethics Committee of the Unilever Shanghai Clinical Research Centre, China and it was conducted in compliance with the Declaration of Helsinki and its subsequent revisions.

Subjects were excluded from the study if they had used anti-dandruff shampoo containing for example, Zinc Pyrithione (ZPT), selenium sulfide or ketoconazole within the last 6 months, were taking anti-inflammatory medication, or had suffered or were currently suffering from any skin condition on head and / or neck (e.g. eczema, psoriasis).

Scalp condition was evaluated using a visual assessment method, resulting in a numerical score, the total weighted head score, or TWHS [35]. This is a whole head assessment of scalp condition based on evidence of dryness/dandruff of the scalp, loose flakes in the hair, and scalp erythema. Subjects were classified into healthy and dandruff groups based on their TWHS in line with previous methodologies [4]. In order to ensure a broad distinction between healthy and dandruff subjects, study participants with a TWHS score of 8 or below were classified as healthy whereas subjects with a TWHS of greater than 32 were classified as dandruff.

All subjects had their scalp condition re-assessed after a 4-week run-in phase during which they washed their hair at home up to 3 times per week with a commercial beauty shampoo which did not contain any antifungal ingredients. Subjects who still met the scalp condition criteria were enrolled onto the study. Subjects that did not meet these criteria were not included in the study.

Scalp sites from which samples were collected were assessed at the point of sampling by a trained scalp assessor, using the grading system described in Table 1. All subjects had two buffer scrub samples collected, one at a site with the highest severity flaking and the other with healthy or lowest grade flaking. Sample groupings were defined as a combination of TWHS grouping and a site score grouping. In this study, 4 sample groupings were used, Healthy_O (Healthy scalp–O Grade Site), Healthy_A (Healthy scalp–A Grade Site), Dandruff_O (Dandruff scalp–O Grade Site), and Dandruff_C (Dandruff scalp–C Grade Site). Groupings were selected in an attempt to ensure differentiation between the healthiest and least healthy site on the scalp to facilitate analysis.

**Table 1:** Definitions for the dandruff grade scoring used to assess severity of scalp dandruff.

Grade	Severity of adherent scalp flaking
**O**	Perfect, healthy scalp. Uniform texture, no surface flakes.
**A**	Minimal dry powdery flakes.
**B**	Small flakes at least partially adhered to the scalp.
**C**	Moderate flakes, loosely attached to the scalp. Scalp surface irregular and white.

In total 65 subjects, male (n = 25, 11 Healthy and 14 Dandruff) and female (n = 40, 19 Healthy and 21 Dandruff) subjects, between the ages of 20–63, completed the study.

### Sample collection

Samples were obtained from the scalp sites using the method of Williamson and Kligman [36]. The method involves placing a sterile Teflon ring of 19mm internal diameter (area = 2.83 cm^2^) over the site to be sampled. Using a sterile plastic disposable pipette, 2.0ml of sterile phosphate buffered saline pH 7.9 (with 0.1% TritonX-100) was applied to the sample site. The scalp surface was then gently agitated with a Teflon rod for one minute. The resulting suspension was transferred to a sterile vial using a sterile plastic Pasteur pipette. This process was repeated at the same sampling site and the samples pooled. Samples were placed on ice during the collection process and then stored at -80°C prior to DNA extraction.

### DNA extraction

All samples were concentrated by centrifugation (10mins/13,000rpm, Eppendorf 5810R, Germany), supernatant removed, and the cells resuspended in 500μl of sterile TE buffer (10 mM Tris-HCl; 1 mM EDTA, pH 7.4). Lysis of cells was carried out by adding 3μl of Ready-Lyse lysozyme (250U/μl) (Epicentre, Wisconsin, USA). Cell suspensions were incubated at 37°C for 18h with agitation at 200rpm, followed by bead beating at 6.5m/s for 2 x 45secs, with 5 minutes rest between bursts, using the FastPrep 24 machine (MPBiomedicals, California, USA). DNA was subsequently extracted and purified from the samples using a DNeasy Blood & Tissue DNA extraction kit (Qiagen, Germany) following the manufacturer’s protocols.

### PCR amplification and 454 sequencing

Bacterial and fungal-derived Polymerase Chain Reaction (PCR) amplicons were produced for each sample using universal primers (Table 2).

**Table 2:** Primers used for amplification of 16S rRNA gene and ITS2 regions.

Primer ID	Target	Primer Sequence
**515f**	16S rRNA gene	5’- GTGCCAGCMGCCGCGGTRA -3’
**1061r**	16S rRNA gene	5’- CRRCACGAGCTGACGAC -3’
**ITS3MF**	ITS2	5’- SCATCGATGAAGAACGCAGC -3’
**ITS4R**	ITS2	5’- TCCTCCGCTTATTGATATGC -3’

For bacteria, an 550bp fragment (V4 –V6) of the 16S rRNA gene was amplified using a primer set previously evaluated *in-silico* using the open-source software PrimerProspector [37]. The forward primer comprised: 454 Life Sciences Adaptor A; primer key “TCAG”; a unique 12-bp error-correcting Golay code used to tag each sample; and the bacterial primer 515f. The reverse primer comprised: 454 Life Sciences Adaptor B; primer key “TCAG”; and bacterial primer 1061r.

For fungi, the ITS2 region of the fungal rRNA gene was also amplified. The forward primer comprised: 454 Life Sciences Adaptor A; primer key “TCAG”; a unique 12-bp error-correcting Golay code used to tag each sample; and the fungal primer ITS4R. The reverse primer comprised: 454 Life Sciences Adaptor B; primer key “TCAG”; and the fungal primer ITS3MF.

PCR reactions for bacteria and fungi were carried out in triplicate and consisted of 1μl of each 5μM primer, 2 μl of 10x CoralLoad Concentrate, 10μl of HotStar Taq Plus Mastermix (Qiagen, Germany), 2 μl of template DNA and 4 μl of molecular grade water (Sigma, USA). The samples were amplified using the following parameters: 95°C for 5 min, 35 cycles of; 95°C for 45s, 55°C for 30s, and 72°C for 60s, with a final extension of 10min at 72°C (PTC-200, Bio-Rad, USA). The replicate PCR reactions from each sample were pooled, visualized by gel electrophoresis (1.0% Biology Grade agarose, Invitrogen, USA) and purified using the DNA extraction kit (AxyPrep DNA Gel Extraction Kit, Axygen, Netherlands) following manufacturer’s instructions. DNA was then further purified by magnetic beads (Agencourt AMPure XP, Beckman Coulter, USA). PCR amplicons from each sample were quantified using Quant-IT Broad Range dsDNA kit (Invitrogen, USA), and 2 composite pools (one bacterial and one fungal) for pyrosequencing were prepared by pooling equimolar concentrations of PCR amplicons.

Samples were sent to the Beijing Genomics Institute (BGI) at Shenzhen, China for pyrosequencing on the Genome Sequencer FLX (Roche 454 Life Sciences, Branford, CT). The pooled amplicons were checked on an Agilent Bioanalyzer DNA HS chip for the absence of primer-dimers and quantitated using the Qubit^®^ dsDNA HS Assay Kit, with the Qubit^®^ 2.0 Fluorometer (Invitrogen). The amplicon libraries were diluted to 1×10^7^molecules/μl. Emulsion PCR was set up according to Roche's recommendations. Following emulsion PCR enrichment, beads produced were deposited into 2-region gasket format wells of a PicoTiterPlate device. 454 sequencing was performed using the GS FLX+ instrument with Titanium XLR chemistry according to the manufacturer’s recommendations. Image analysis, shotgun signal processing, and base calling were performed using the supplied system software (version 2.6). The Standard Flowgram Format (.sff) files output from base calling were employed in subsequent analysis. All sequencing data has been deposited in the SRA (Accession number SRP148955).

### Informatics processing

Following amplicon sequencing, resulting flowgrams were processed using the AmpliconNoise algorithm [38] to remove inherent noise in the data which was shown previously to drastically over-estimate true levels of α-diversity [39]. Chimeras were removed using the AmpliconNoise associated Perseus algorithm, and the resulting denoised sequences were clustered into Operational Taxonomic Units (OTUs) using Vsearch [40] v1.9.6, with a cluster identity of 0.97 and a minimum cluster size of 10.

Bacterial OTUs were taxonomically classified against the SILVA [41], NCBI [42], RDP [43], DDBJ [44], Greengenes [45], CAMERA [46], EzBioCloud [47], and EMBL [48] databases using a Lowest Common Ancestor (LCA) methodology. For taxonomic assignment to be made, at least 50% of the data sources queried had to classify the OTU to the same level of phylogeny (species, genus, order etc.). Fungal OTUs were assigned using the same LCA methodology against SILVA, NCBI, UNITE [49], CAMERA, DDBJ, and EMBL databases.

The OTU table and associated representative sequences, selected as the most abundant sequence within the OTU cluster, were used as inputs for QIIME [50] version 1.9.1 (Quantitative Insights into Microbial Ecology), an open source software package for analysis of complex microbial communities.

### PCR amplification and sanger sequencing

Speciation of *Staphylococcus* species was facilitated by Sanger sequencing of selected samples. For each sample, a region of the *tuf* and *gap* genes were amplified using a primer set designed in-house (Table 3). PCR reactions were performed as described above.

**Table 3:** Forward (F) and reverse (R) primers designed for amplification of *tuf* and *gap* genes from *Staphylococcus* taxa.

Taxa	Target gene	Sequence (5’-3’)
***Staphylococcus* spp.**	*tuf*	tuf338F: 5’- TGCCACAAACTCGTGAACACA -3’
		tuf693R: 5’- ACTTTGATTTGACCACGTTCAACA -3’
	*gap*	gap144F19: 5’- YACTATGCAAGGWCGYTTC -3’
		gap674R21: 5’- CSCCACCRTCTAATTTACCRT -3’

The PCR products were separated with 1.5% agarose gel electrophoresis, and the target fragments were retrieved and purified by Axygen Agarose Gel DNA Purification Kit (Axygen Netherlands). The target fragments were ligated to pMD ^™^19 -T using the T-vector Cloning Kit (Takara, China). The recombinant pMD19-T-*tuf* or pMD19-T-*gap* constructs were sent to Life technologies for sequencing using an ABI 377 DNA sequencer (Thermo Fisher Scientific, USA).

### Quantitative PCR (qPCR)

Amplification primers were designed and optimised. Probes were labelled with the fluorescent reporter dye 6-FAM (Fluorescein) at the 5’ end and BHQ1 as a quencher at the 3’ end. Primers and probes are listed in Table 4.

**Table 4:** Sequences of qPCR primers and probes for the quantification of bacterial and fungal taxa.

Taxa	Target	Sequence (5’-3’)
***M*. *globosa***	ITS2	M.g13F19: 5’- AAACAGCTAACGCCTCTGG -3’
		M.g88R21: 5’- ATGACGTATCATGCTATGCCT -3’
		M. g32P22: 5’- CTGGGCCACTTTGCATCCGCTT -3’
***M*. *restricta***	ITS2	M.r86F19: 5’- CTCTGCCTGCGCTACCTAG -3’
		M.r143R21: 5’- AAACAGAGAGGCGGATGCAAA -3’
		M.r108P21: 5’- AGGCTCGCCCGAAATGCATGA -3’
***Staphylococcus* spp.**	16S	St110F24: 5’- GGGTGAGTAACACGTGGATAACCT -3’
		St250R24: 5’- GCGGCGCGGATCCATCTATAAGTG -3’
		St141P28: 5’- GACTGGGATAACTTCGGGAAACCGGAG -3’
***C*. *acnes***	16S	PA1-129F: 5’- GACTTTGGGATAACTTCAGGAAACTG -3’
		PA1-238R: 5’- CTGATAAGCCGCGAGTCCAT -3’
		PA1-195P: 5’- TTGGAAAGTTTCGGCGGTTG -3’
***S*. *epidermidis***	*atlE*	atlE_1610F: 5’- GGAGGAACTAATAATAAGTTAACTG -3’
		atlE_1702R: 5’- GTCATAAACAGTTGTATATAAGCC -3’
		atlE_1638P: 5’- CTGCTAATCGTGGTGTTGCTCAAATTAAA -3’
***S*. *capitis***	*gap*	gap1_179F20: 5’- GAGACCTAGACATCGACGTA -3’
		gap1_616R18: 5’- AACTGGAACACGTTGAGCTC -3’
		gap_478U24: 5’- CTCAAGATGCTCCTCACAGAAAAG -3’

qPCR reactions comprised 10μl Premix Ex Taq^™^ (Takara), 2μl of each 5μM primer mix (Thermo Fisher Scientific, USA), and 2μl of a 10μM probe (Thermo Fisher Scientific, USA), with the volume made to 20μl with molecular biology grade water. Triplicate test samples of DNA (2μl) or dilutions of DNA standard were added to a 96-well assay plate (Axygen, Netherlands). The plates were sealed with a clear plastic adhesive sheet (ABI), placed in an ABI 7500 real-time PCR machine with an initial denaturation at 94°C, for 2mins and cycled 40 times (94°C, 15s; 60°C, 1min). Data was collected, and copy number calculated by the ABI 7500 software.

### Phylogenetic visualisation

Phylogenetic trees were produced using Phylogeny.fr [51] and TreeDyn [52]. The horizontal lines represent the branching of evolutionary lineages over time, with a longer line representing a greater degree of genetic variation. The bottom bar offers a scale for this genetic variation in the form of the length of branch that represents a genetic change of 0.07 (or 7 nucleotide substitutions per 100 bases). Sites of branching represent species genetically diverging from each other. The number shown in red next to each node represents the level of support for that node, where 1 represents maximal support. A higher value suggests strong evidence that those sequences to the right of that node cluster together to the exclusion of any of the others.

Pairwise alignment plots were produced using Sequence Demarcation Tool (SDT) v1.2 [53]. Using a FASTA input file SDT aligns every unique pair of sequences, calculates pairwise similarity scores, and displays a colour coded matrix of these scores. The identity scores are calculated as 1-(M/N) where M is the number of mismatching nucleotides and N the total number of positions along the alignment at which neither sequence has a gap character.

### Statistical analyses

#### Metataxonomics

Statistical analyses were performed on the table of counts produced from the bioinformatics pipeline. Sample library sizes were first normalised at OTU level using rarefaction, 3300 reads per sample for bacteria and 1000 reads per sample for fungi. The level was chosen to preserve as many samples as possible while ensuring sampling integrity. This resulted in the following sample groups (Table 5).

**Table 5:** Number of samples in each group (scalp type and site score) remaining after rarefaction processing.

Scalp Type	Site Score	N—Fungal	N—Bacterial
**Dandruff**	C	34	31
	O	34	31
**Healthy**	A	29	23
	O	29	25

The rarefied OTU tables were further aggregated to genus level and analyses performed for both. Sample and group beta diversity was visualised using a Bray-Curtis dissimilarity with non-metric multidimensional scaling. The Bray-Curtis distance is a semi-metric distance commonly used in microbial ecology and in the form employed accounts for both differences in species composition and their mean relative abundance. Analysis of variance between scalp types and site score classes was performed using permutation analysis of variance (PEMANOVA) on the Bray-Curtis dissimilarity matrix. Analyses were performed using the R packages Vegan [54], Party [55] and HMP [56] with q-value to control for false discovery [56].

#### qPCR

The qPCR measurements were made in triplicate and these were summarised using the median value to provide one data point per subject/site. The qPCR copies/ml data was log transformed prior to statistical analysis. Because of the presence of zeros, a value of 1 was added onto every observation prior to log transformation.

Mixed models were used to analyse data with separate models for each species. TWHS/site grade were included as fixed effects and the subject was included as a random effect. Models were run in JMP^®^, Version V10.0.2. SAS Institute Inc., Cary, NC, 1989–2007 [57].

## Results

### Part I. Taxonomic classification of the scalp mycobiome on healthy and dandruff subjects

Following data processing as described earlier, a dataset consisting of 860,030 high quality partial ITS2 sequences was obtained. One hundred and thirty samples with an average of 6467 sequences per sample were analysed. Clustering and classification were performed using the LCA methodologies described above and resulted in 273 operational taxonomic units (OTUs) which collapsed to 86 genera.

#### Association of the scalp mycobiome with scalp condition

**Fig 1:**
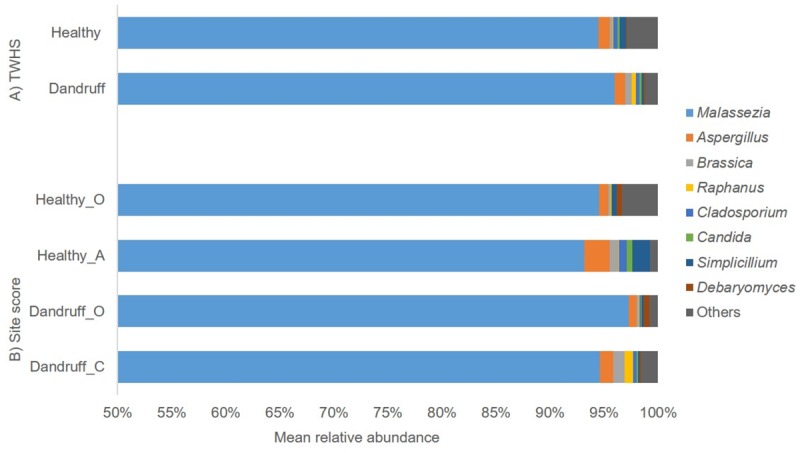
Fungal mean relative abundance. Mean relative abundance of the predominant fungal genera as assessed by: A) TWHS: (Healthy and Dandruff) and B) Site score: Healthy (O and A) and Dandruff (O and C). The y-axis is truncated at 50% to facilitate visualisation of small changes in relative abundance.

The eight most abundant taxa observed in the scalp mycobiome are shown in Fig 1. Analysis was split based on TWHS (A) and TWHS plus individual site score (B). The most abundant genus level taxon was *Malassezia* accounting for >86% of the taxa observed across all samples (data not shown). No significant difference in *Malassezia* spp. relative abundance was found when samples were graded by TWHS (Fig 1A; healthy, 94.48%; dandruff, 95.97%) or by individual site score (Fig 1B).

**Fig 2:**
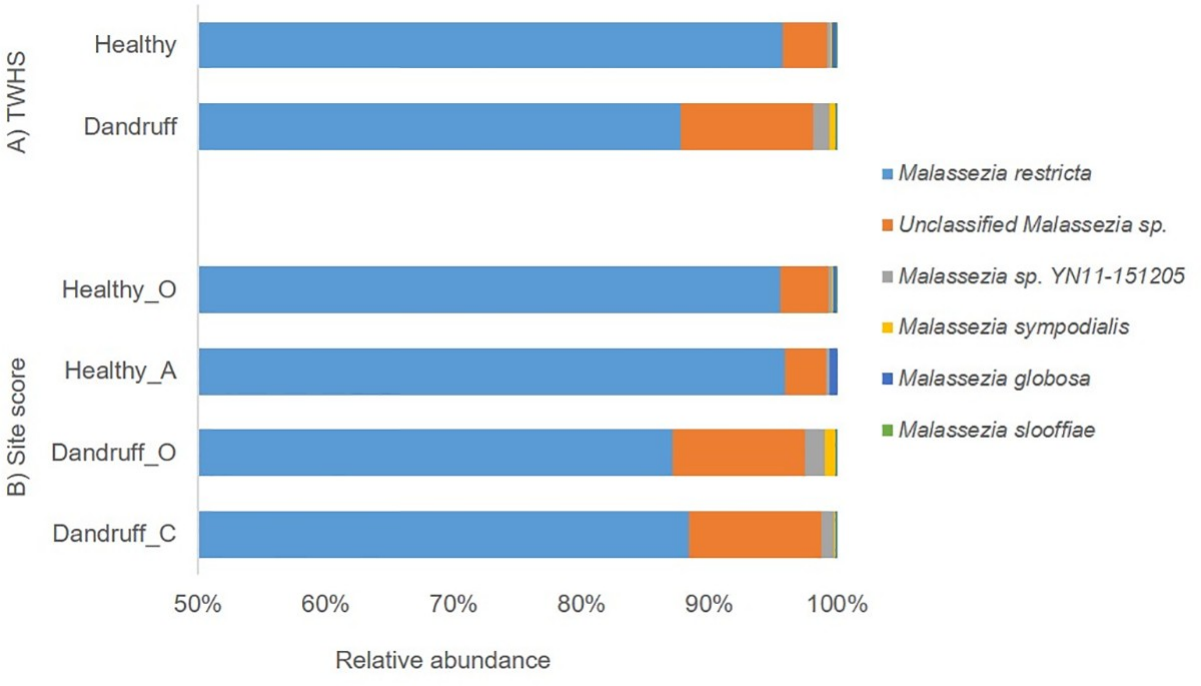
*Malassezia* spp. mean relative abundance. Mean relative abundance of *Malassezia* taxa as assessed by: A) TWHS: Healthy and Dandruff and B) Site score: Healthy (O and A) and Dandruff (O and C). The y-axis is truncated at 50% to facilitate visualisation of small changes in relative abundance.

Analyses of the *Malassezia* taxa at species level showed the presence of 6 taxa and revealed the dominance of *M*. *restricta* across all sites sampled. It was not possible to unequivocally identify the second most abundant taxon labelled as unclassified *Malassezia* sp. (Fig 2), which was found to be present at a significantly higher relative abundance (p = 0.0467) in the dandruff group (9.91%) compared with healthy (3.30%). Subsequent analysis, described later, suggests that this taxon is a previously undescribed member of the *Malassezia* genus.

#### Quantitative assessment of the scalp mycobiome on healthy and dandruff scalps

**Fig 3:**
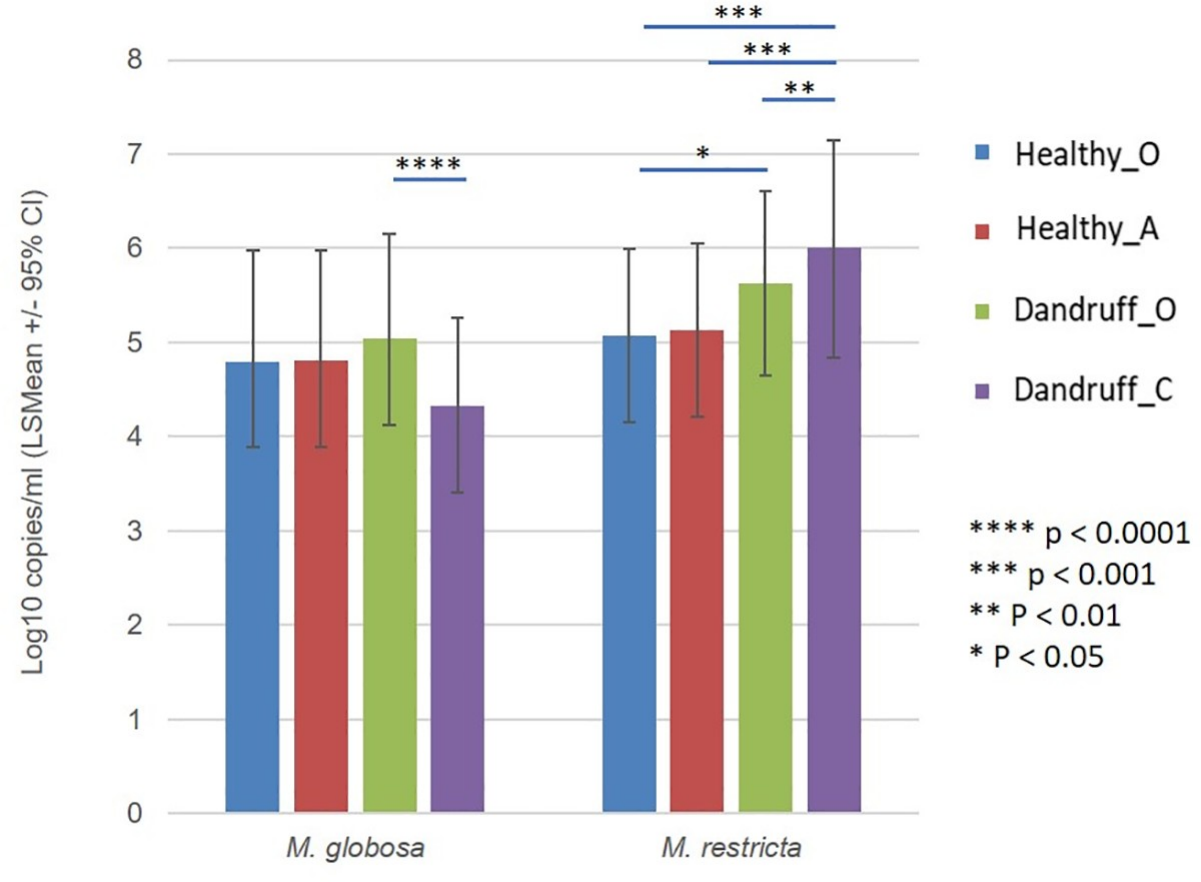
*Malassezia* enumeration by qPCR. Least Square Means (LSMean) log 10 copies/ml for *M*. *globosa* and *M*. *restricta* at different scalp sites graded on the basis of their dandruff severity: Healthy_O site; Healthy_A site.; Dandruff_O site; and Dandruff_C site. Statistically significant differences between groups are highlighted by means of connecting bars with associated p values.

Metataxonomics provides information on the relative abundance of microbial community members. While this is informative, qPCR assessment of absolute microbial abundance of taxa of interest is necessary to indicate associations between target taxa and the condition being examined. qPCR was carried out for representative scalp *Malassezia* species, namely *M*. *globosa* and *M*. *restricta* (Fig 3). The absolute abundance of *M*. *globosa* was similar for all sites bar the most severe flaking site (Dandruff_C), where this was found to be significantly lower when compared with healthy sites (Dandruff_O) from the same individuals (p = <0.0001). Conversely a significant increase was observed in *M*. *restricta* for the most severe flaking site, Dandruff_C, compared with healthy sites obtained from the same individuals, Dandruff_O (p = 0.0047) and when compared with Healthy_O (p = 0.0004) and Healthy_A, (p = 0.0011) sites.

#### Examination of the unidentified fungal taxa

**Fig 4:**
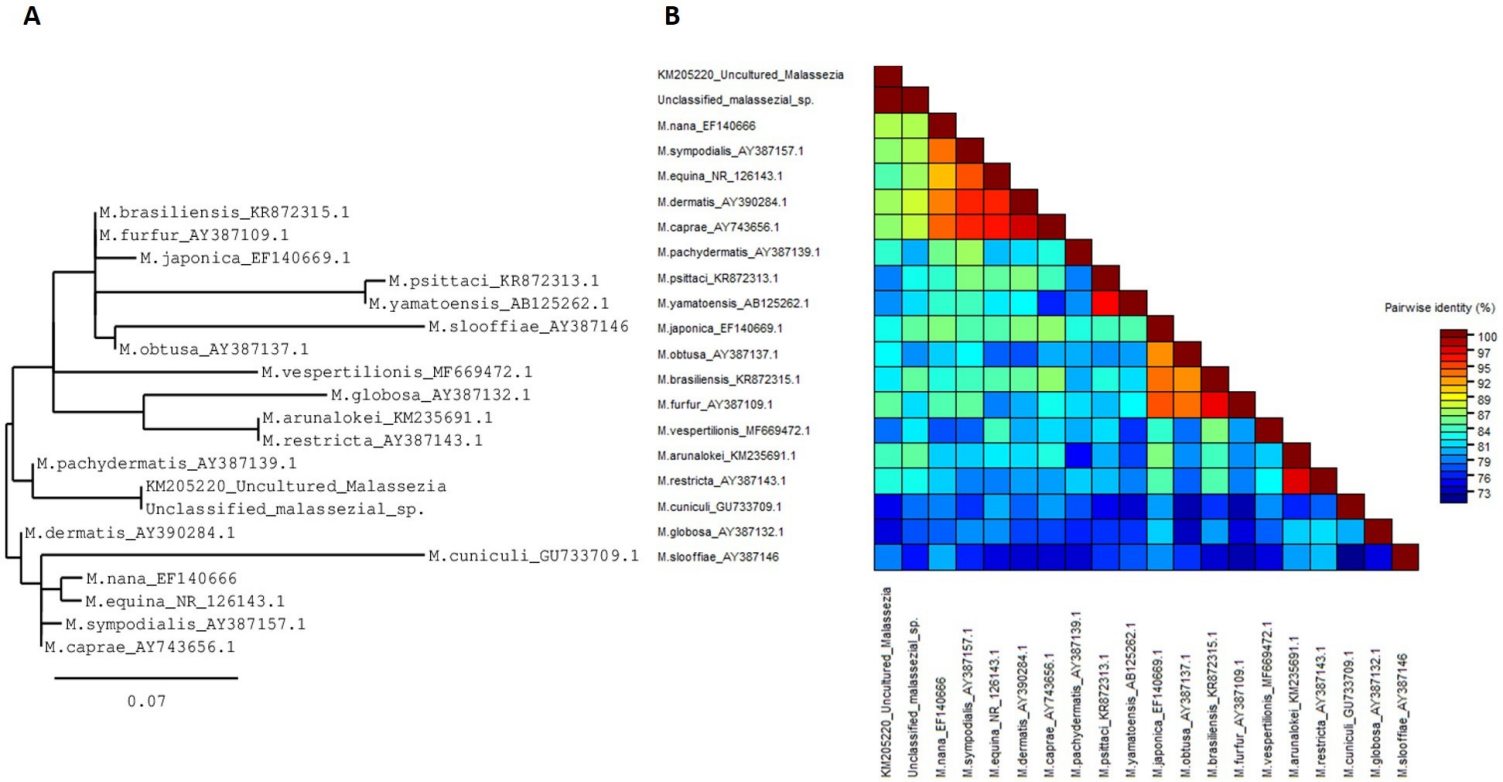
Phylogenetic assessment of *Malassezia* species. (A) Phylogenetic tree showing the evolutionary relationship across 423 bases of the ITS2 region for the 18 described species of *Malassezia*, *Malassezia* KM205220 reported by Soares et al, and the unclassified *Malassezia* sp. reported here. (B) Pairwise alignment plot showing pairwise identity of the 20 sequences.

Metataxonomic analysis of the scalp mycobiome revealed the presence of an abundant but unclassifiable fungal taxon. Sequence similarity analysis of this OTU suggests that this taxon belongs to the *Malassezia* genus (Fig 4). The representative sequence from this OTU was subsequently compared with sequences from all 18 currently reported *Malassezia* species. ITS2 sequences, and an ITS2 sequence (KM205220) proposed by Soares *et al*. [58] as representing a new *Malassezia* species. Fig 4A shows the results of this analysis as a phylogenetic tree. The unclassified OTU sequence and KM205220 cluster together and this node is discrete from other *Malassezia*. The tree shows a unique pattern not seen before in comparisons of *Malassezia*, most likely due to the specific region 423bp region of ITS2 sequence available for full alignment. Pairwise alignment (Fig 4B) confirmed that this unclassified *Malassezia* sp. has 100% sequence identity with the unclassified, proposed *Malassezia* sequence, plus further unclassified *Malassezia* OTUs found previously in two Unilever UK studies (unpublished data).

The unclassified, proposed *Malassezia* sp. shared limited similarity over the 423 bases of the ITS2 sequence that were available for alignment, with representative sequences of *M*. *globosa* (77.7%), *M*. *restricta* (83.1%), *M*. *sympodialis* (87.4%), *M*. *slooffiae* (75.9%), *M*. *nana* (87.3%) and *M*. *pachydermatis* (80.5%). In general, the unclassified *Malassezia* sp. has between 75.9% and 88% similarity with known *Malassezia* spp.

### Part II. Taxonomic classification of the scalp bacterial microbiome on healthy and dandruff subjects

Following data processing as described earlier, a dataset consisting of 1,097,397 high-quality 16S rRNA partial gene sequences was obtained. Fourteen samples were removed following AmpliconNoise processing due to low quality data. The remaining 116 samples had an average of 9,303 sequences per sample. Clustering and LCA classification resulted in 300 operational taxonomic units (OTUs) which collapsed to 114 genera and an unclassified taxon. Taxa were determined at the ≥97% sequence similarity level.

#### Association of the scalp bacterial microbiome with scalp condition

**Fig 5:**
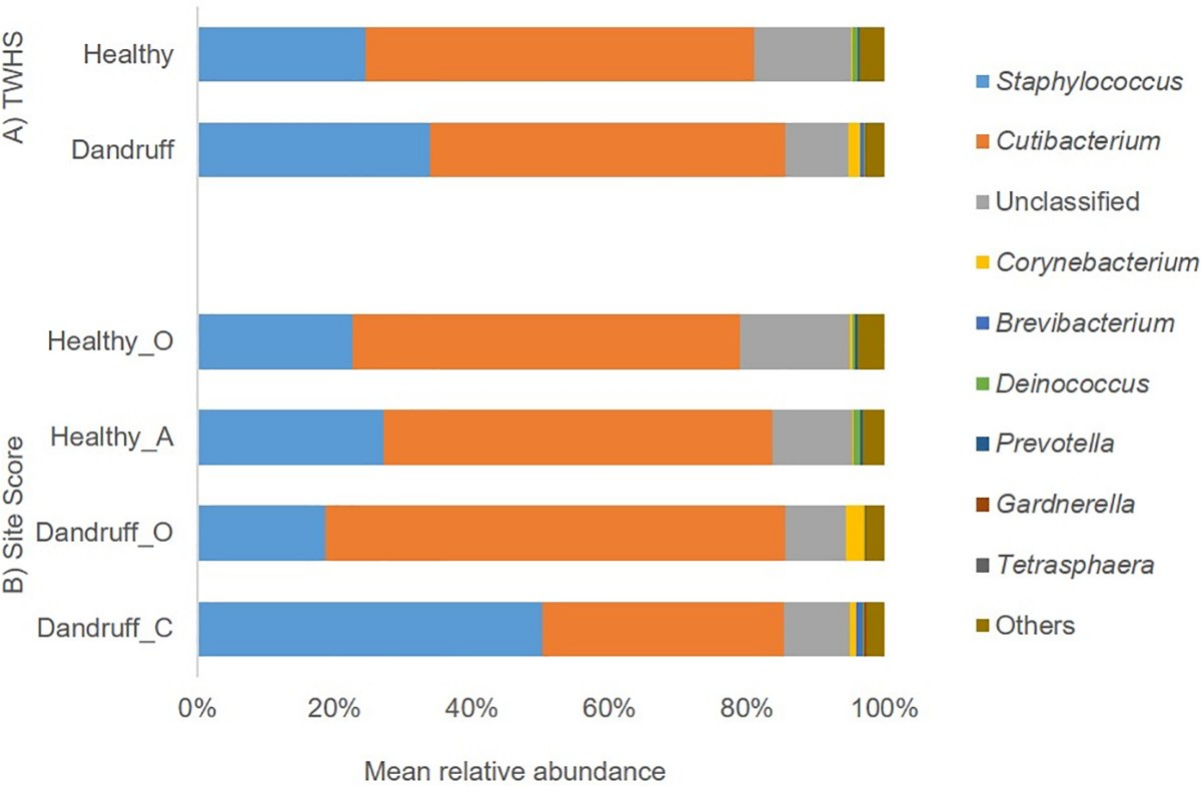
Bacterial mean relative abundance. Mean relative abundance of the predominant bacterial genera as assessed by: A) TWHS (Healthy and Dandruff) and B) Site score: Healthy (O and A) and Dandruff (O and C).

The most abundant genera observed across all samples were *Staphylococcus* and *Cutibacterium*. The mean relative abundance of *Staphylococcus* spp. based on TWHS (Fig 5A) was 24.5% in healthy subjects and 33.7% in dandruff subjects. A decrease in mean relative abundance of *Cutibacterium* from 56.2% in healthy subjects to 51.5% in dandruff subjects was observed. In both of these instances the changes in relative abundance for these genera between healthy and dandruff groups, based on TWHS, were not significant.

The most abundant taxa observed when samples were split by site score are shown in Fig 5B. A statistically significant increase in the relative abundance of *Staphylococcus* spp. was observed in samples taken from Dandruff_C sites compared with Dandruff_O or Healthy_O sites (p <0.0001). Conversely, there was a statistically significant decrease (p<0.0001 & p = 0.0064 respectively) in the relative abundance of *Cutibacterium* when comparing these same sites.

#### qPCR assessment of *C*. *acnes* and *Staphylococcus* spp. in healthy and dandruff subjects

**Fig 6:**
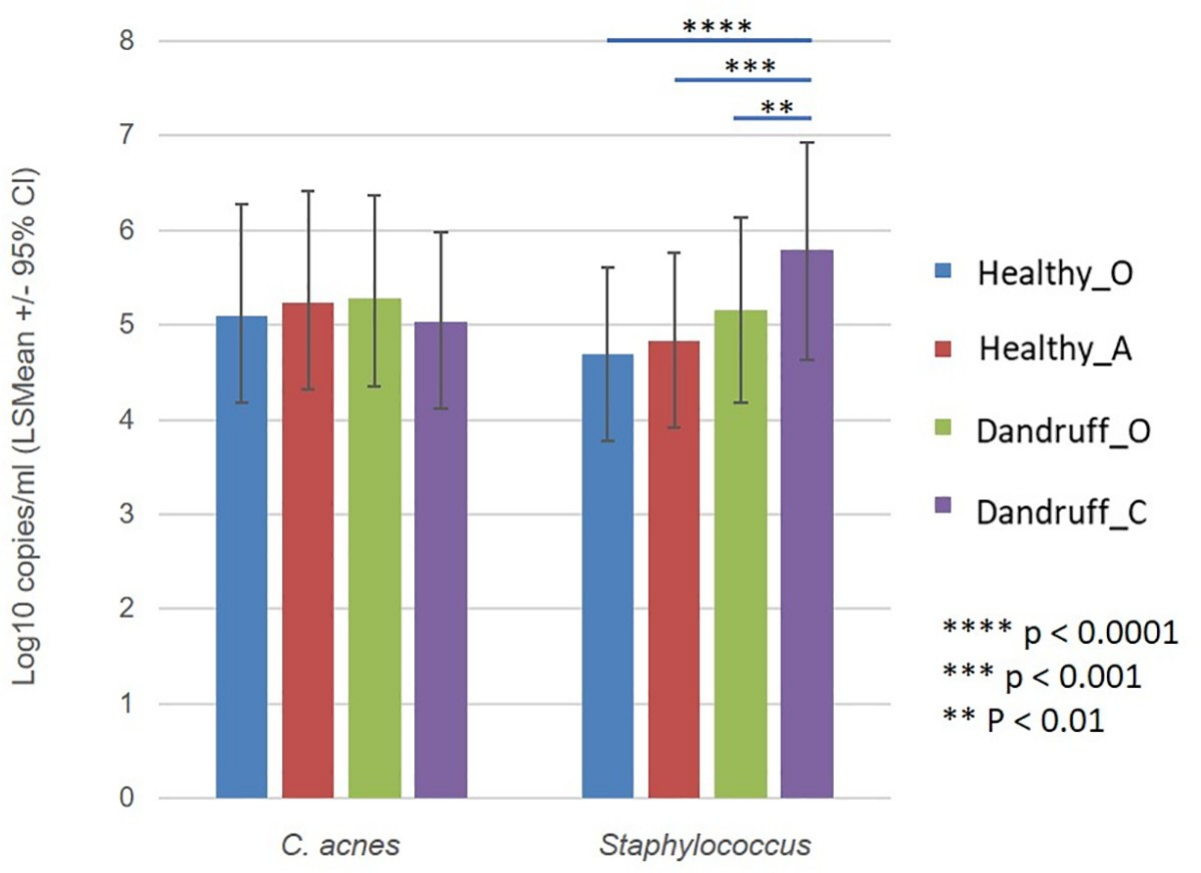
Bacterial enumeration by qPCR. Least Square Means (LSMean) log 10 copies/ml for *C*. *acnes* and *Staphylococcus* spp. at different scalp sites graded on the basis of their dandruff severity: Healthy_O site; Healthy_A site.; Dandruff_O site; and Dandruff_C site. Statistically significant differences between groups are highlighted by means of connecting bars with associated p values.

Site score based qPCR evaluation was carried out on all samples at species level for *C*. *acnes* and at genus level for *Staphylococcus*. The results of these assays are shown in Fig 6. No significant associations were seen between *C*. *acnes* absolute abundance and scalp site score in the study subjects. There was however a significant increase in *Staphylococcus* spp. absolute abundance at Dandruff_C sites when compared with all other sites (p ≤0.0013). All statistically significant differences can be found in the supplementary data (S1 Table).

#### Speciation of the predominant *Staphylococcus* species in the scalp microbiome

The human skin microbiome is known to include multiple species of the genus *Staphylococcus* [59]. Due to the increase in both the relative and absolute abundance of staphylococci found on dandruff lesional sites it was desirable to attempt to speciate the members of this genus. Primers targeting the *gap* and *tuf* genes, commonly used to speciate staphylococci [60], were designed to facilitate amplification, cloning and Sanger sequencing of these genes from the study samples. Sequencing of individual clones identified the presence of both *S*. *capitis* and *S*. *epidermidis* in all samples examined.

#### qPCR assessment of *S*. *capitis and S*. *epidermidis* in healthy and dandruff scalps

**Fig 7:**
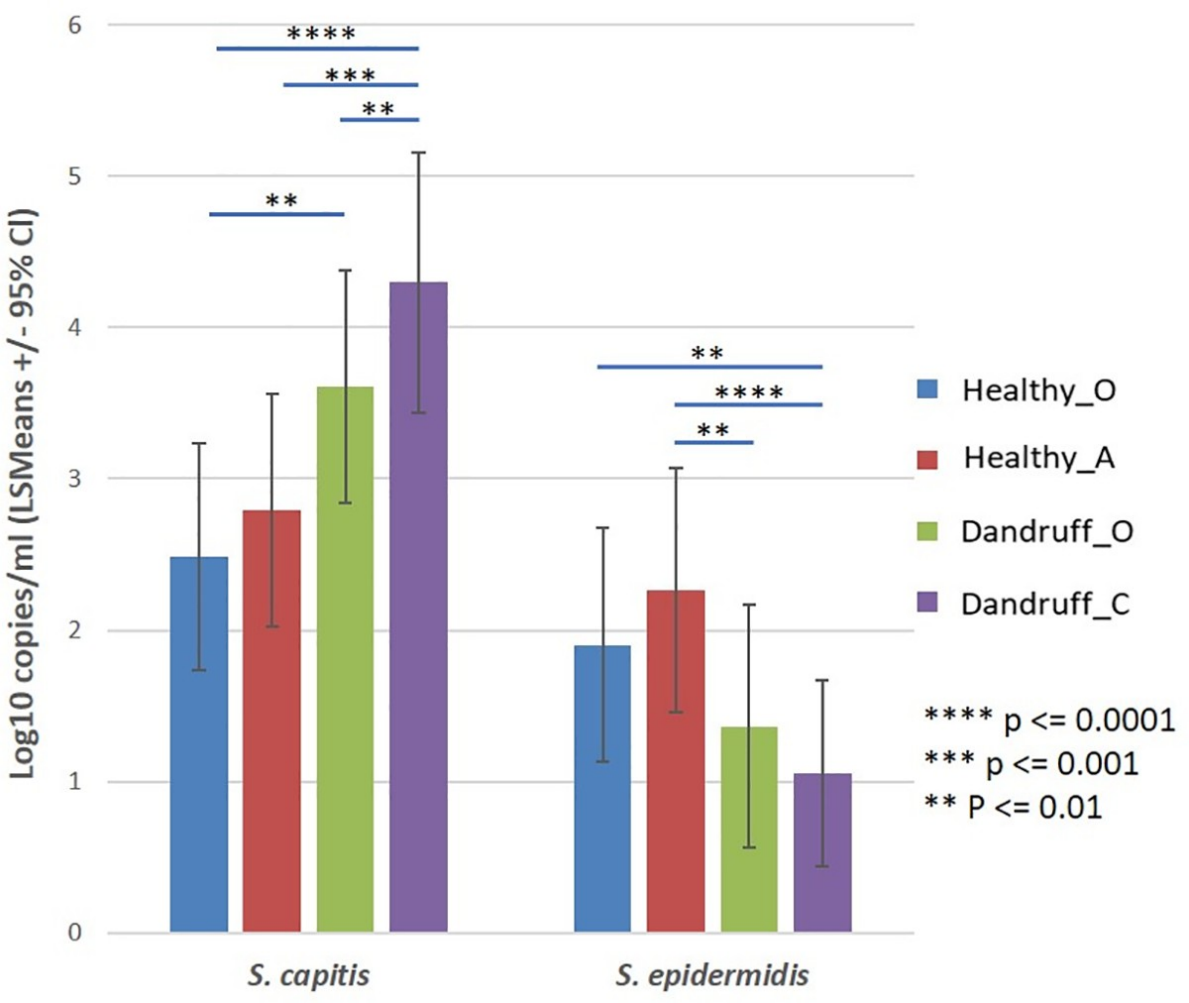
*Staphylococcus* species enumeration by qPCR. Least Square Means (LSMean) log 10 copies/ml for *S*. *capitis* and *S*. *epidermidis* at different scalp sites graded on the basis of their dandruff severity: Healthy_O site; Healthy_A site.; Dandruff_O site; and Dandruff_C site. Statistically significant differences between groups are highlighted by means of connecting bars with associated p values.

qPCR assays were designed and implemented to quantify the levels of both *S*. *capitis and S*. *epidermidis* in healthy and dandruff scalps. *S*. *capitis* was quantified by assessment of the *gap* gene *S*. *epidermidis* was quantified by assessment of *atlE* gene [61, 62]. *S*. *capitis* was determined to be the most abundant *Staphylococcus* species in scalp samples from both healthy and dandruff scalps (Fig 7). qPCR analysis showed that *S*. *capitis* levels increased in line with an increase in site score representing a statistically significant increase in absolute abundance between Healthy_A/Healthy_O and Dandruff_C sites (p = 0.0009 and p<0.0001 respectively) and Ddandruff_0 and Healthy_O sites (p = 0.0112).

qPCR analysis of *S*. *epidermidis* showed an overall decrease in absolute abundance concomitant with an increase in site score, representing a statistically significant decrease in the abundance of *S*. *epidermidis* between Healthy_A/Healthy_O and Dandruff_C sites (p≤0.0048). All statistically significant differences can be found in the supplementary data (S1).

#### Site score correlates with bacterial community composition

**Fig 8:**
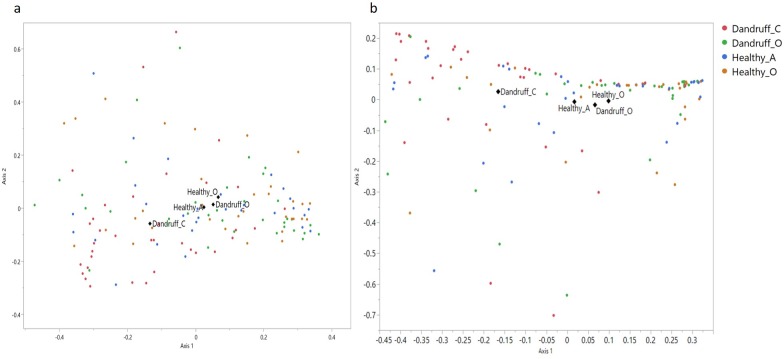
**Non-metric Multi-Dimensional Scaling Bray Curtis analysis of bacterial communities at genus (a) and OTU (b) level at differentially graded scalp sites.** Beta Diversity analysis of bacterial communities shows significant differences (p>0.01), at both genus and OTU level, between lesional sites on a dandruff scalp (Dandruff_C) and all other sites tested. Non–lesional sites on dandruff scalps (Dandruff_O) cluster more closely with sites from a healthy scalp (Healthy_O and Healthy_A).

Bacterial and fungal communities at all sites were assessed via nonmetric multidimensional scaling (NMDS) at genus and OTU level to examine relationships between community composition and TWHS/site score. Analysis of fungal communities did not reveal any distinct community clustering based on TWHS or site score. Analysis of bacterial communities however showed that samples from both sites on healthy scalps (Healthy_O and Healthy_A) clustered together with samples from non-lesional sites on dandruff scalps (Dandruff_O) with all three clustering away from lesional sites on dandruff scalps (Dandruff_C). Statistical analysis showed that Dandruff_C sites were statistically different to all other sites (p < 0.001), Fig 8. These results are at odds with some previous observations where dysbiotic communities in dandruff were seen to be present in non-clinically affected sites [63]. However, the data presented here suggests that localised microbial dysbiosis is occurring at lesional sites only and is consistent with previous host response analysis showing that human transcriptomics response in the dandruff condition is also localised to lesional sites [64].

## Discussion

The work described here adds to previous studies characterising the fungal mycobiome and bacterial microbiome of healthy and dandruff afflicted scalps [16, 17, 34]. Importantly, this study adds an additional detailed focus on the differentiation of both healthy and dandruff scalps by site score with associated qPCR analysis for the key microbial species. This more focused investigation allows for both intra- and inter-scalp comparisons utilising differentially graded sites for each healthy or dandruff scalp type.

As demonstrated previously and irrespective of scalp condition, the major component of the fungal mycobiome of both healthy and dandruff scalps is *Malassezia restricta*. The results presented here are in agreement with a long association of the dandruff condition with an alteration in the abundance of *M*. *restricta*. While no differences were seen in the relative abundance of *M*. *restricta* and *M*. *globosa*, significant differences in absolute abundance (qPCR) for both *Malassezia* species were evident when comparing lesional and non-lesional sites on healthy and dandruff scalps. These results demonstrate that comparative *Malassezia* species abundance on lesional versus non-lesional sites on a dandruff scalp differ more significantly than the same comparisons on the healthiest versus the least healthy sites on a healthy scalp. This opens up an intriguing possibility of not only individual host susceptibility [29], but also the potential for localised host susceptibility which facilitates the localised alteration of *Malassezia* spp. abundance.

Metataxonomic analysis identified scalp-associated members of the *Malassezia* genus including *M*. *restricta*, *M*. *globosa* and *M*. *sympodialis*. In addition, an OTU previously classified as an uncultured *Malassezia* sp. was present in samples from both healthy and dandruff scalps. Notably this OTU was present at a significantly higher abundance than the more commonly identified *M*. *globosa*. This OTU matches a previously described uncultured *Malassezia* species identified in a Brazilian cohort of seborrheic dermatitis patients [58]. Since the Soares *et al* work focused on *Malassezia* species diversity assessment, no correlation was drawn between the presence of this species and instances of seborrheic dermatitis. In this present study however, the relative abundance of this uncultured species was significantly higher on dandruff scalps when compared with healthy (p = 0.0467). While this is insufficient evidence to suggest causation, these results indicate that both quantitative assessment, isolation and characterisation of this taxon is warranted in future studies. Full genomic analysis will be necessary to determine the phylogenetic relationship of this species with others previously characterised [12]. In the course of this work multiple attempts were made to culture the uncultured *Malassezia* species. A species-specific qPCR assay to enumerate the uncultured *Malassezia* species was developed and deployed on a panel of subjects with and without dandruff. Multiple individuals were identified for culture analysis where levels of the uncultured *Malassezia* species where greater than *M*. *restricta*. To date, despite utilising multiple standard and enriched *Malassezia* culture media, it has not been possible to isolate a viable culture of this novel species. Recently additional *Malassezia* species have been discovered across multiple hosts and geographies facilitated by the use of molecular biology methods [13, 58, 65]. Of particular relevance to this work Saxena et al [65] identified a highly abundant uncultured *Malassezia* species present on both healthy and dandruff affected scalps in an Indian population. Unfortunately, direct comparisons of these species is not currently possible due to different ITS regions being used to characterise the scalp mycobiome. These findings however highlight the necessity for additional focus to be placed on the culture of skin relevant *Malassezia* to facilitate genomic analysis. Such methods should encompass single cell sorting as well as attempts to assemble metagenome assembled genomes from shotgun metagenomics data in an effort to identify metabolic dependencies based on assembled genome content [66].

While perturbations of the scalp fungal mycobiome are more commonly associated with dandruff aetiology, recent studies have suggested a possible contribution from dysbiosis of the scalp bacterial microbiome [16, 17, 34]. The results presented here are broadly consistent with those previously described, with *Staphylococcus* and *Cutibacterium* the most abundant genera across both healthy and dandruff scalps. We have demonstrated that bacterial community composition specifically at dandruff lesional sites is significantly different to, not only sites on a healthy scalp, but also adjacent non-lesional sites on a dandruff scalp. This suggests localised microbial dysbiosis and host response rather than a systemic disruption of microbial growth or host gene expression.

This study is the first to quantitatively enumerate the bacterial species present on the scalp, namely *C*. *acnes*, *S*. *epidermidis* and *S*. *capitis*. Quantitative assessment of bacterial species from both healthy and dandruff scalps revealed differences in the absolute abundance of specific bacterial taxa. The abundance of *S*. *epidermidis* was decreased on dandruff scalps compared with healthy, with a concomitant and significant increase in the levels of *S*. *capitis*. The increase in levels of *S*. *capitis* was of sufficient magnitude to drive an overall increase in absolute *Staphylococcus* species abundance at a dandruff lesional site. The reason for this dramatic shift in levels of both *S*. *epidermidis* and *S*. *capitis* is currently unknown and warrants further study. It can be hypothesised that the increase in the absolute abundance of both *M*. *restricta* and *S*. *capitis* on dandruff scalps are linked with both species potentially having the ability to exploit the changing host physiological conditions, including an increase in scalp barrier disruption and transepidermal water loss, and altered sebum concentration [9].

As well as adding to our previous knowledge on the mycobiome and microbiome composition of healthy and dandruff scalps, this study has shown the importance of being able to move beyond genus level analysis of bacteria as well as the importance of quantitative assessment of bacterial and fungal species. Without these approaches the new insights described here, including the significant increase in the absolute abundance of *S*. *capitis* at dandruff lesional sites, would not have been highlighted. Further investigation of *S*. *capitis*, its potential causative role in the progression of dandruff and its interactions with other members of the scalp microbial community are warranted.

## Supporting information

S1 Table Statistical analysis of dominant fungal and bacterial taxa. Statistically significant differences by taxa and TWHS/site score for A) *M*. *globosa*; B) *M*. *restricta*; C) *Staphylococcus* spp.; D) *S*. *capitis*; E) *S*. *epidermidis*. Statistical differences are indicated by a * next to the p-value.
(DOCX)
