# Supplementary material for: The diversity and abundance of fungi and bacteria on the healthy and dandruff affected human scalp
Source: PLoS One. 2019 Dec 18;14(12):e0225796. doi: 10.1371/journal.pone.0225796 (PMC6919596; doi:10.1371/journal.pone.0225796)
Supplement: S1 Table — Statistically significant differences by taxa and TWHS/site score for A) M. globosa; B) M. restricta; C) Staphylococcus spp.; D) S. capitis; E) S. epidermidis. Statistical differences are indicated by a * next to the p-value. (DOCX) [file pone.0225796.s001.docx]

**S1. Statistical analysis of dominant fungal and bacterial taxa.** Statistically significant differences by taxa and TWHS/site score for A) *M. globosa*; B) *M. restricta*; C) *Staphylococcus* spp.; D) *S. capitis*; E) *S. epidermidis*. Statistical differences are indicated by a * next to the p-value.

No statistically significant differences were found for *C. acnes* therefore a table was not included for this taxa.

**A)*M. globosa***

| **Level** | **- Level** | **Difference** | **Std Err Dif** | **Lower CL** | **Upper CL** | **p-Value** |
| --- | --- | --- | --- | --- | --- | --- |
| DDF_O | DDF_C | 0.723 | 0.154 | 0.417 | 1.029 | <.0001* |
| Healthy_A | DDF_C | 0.477 | 0.286 | -0.088 | 1.043 | 0.0976 |
| Healthy_O | DDF_C | 0.474 | 0.283 | -0.085 | 1.033 | 0.0958 |
| DDF_O | Healthy_O | 0.249 | 0.283 | -0.310 | 0.808 | 0.3804 |
| DDF_O | Healthy_A | 0.246 | 0.286 | -0.320 | 0.811 | 0.3921 |
| Healthy_A | Healthy_O | 0.003 | 0.172 | -0.337 | 0.343 | 0.9856 |

**B)*M. restricta***

| **Level** | **- Level** | **Difference** | **Std Err Dif** | **Lower CL** | **Upper CL** | **p-Value** |
| --- | --- | --- | --- | --- | --- | --- |
| DDF_C | Healthy_O | 0.944 | 0.260 | 0.430 | 1.458 | 0.0004* |
| DDF_C | Healthy_A | 0.879 | 0.263 | 0.358 | 1.400 | 0.0011* |
| DDF_O | Healthy_O | 0.565 | 0.260 | 0.051 | 1.079 | 0.0315* |
| DDF_O | Healthy_A | 0.500 | 0.263 | -0.021 | 1.020 | 0.0599 |
| DDF_C | DDF_O | 0.380 | 0.131 | 0.119 | 0.640 | 0.0047* |
| Healthy_A | Healthy_O | 0.065 | 0.149 | -0.231 | 0.361 | 0.6622 |

**C)S*taphylococcus* spp.**

| **Level** | **- Level** | **Difference** | **Std Err Dif** | **Lower CL** | **Upper CL** | **p-Value** |
| --- | --- | --- | --- | --- | --- | --- |
| DDF_C | Healthy_O | 1.104 | 0.277 | 0.557 | 1.651 | 0.0001* |
| DDF_C | Healthy_A | 0.955 | 0.283 | 0.397 | 1.513 | 0.0009* |
| DDF_C | DDF_O | 0.633 | 0.191 | 0.255 | 1.012 | 0.0013* |
| DDF_O | Healthy_O | 0.471 | 0.277 | -0.076 | 1.017 | 0.0911 |
| DDF_O | Healthy_A | 0.322 | 0.283 | -0.236 | 0.880 | 0.2563 |
| Healthy_A | Healthy_O | 0.149 | 0.211 | -0.270 | 0.568 | 0.4829 |

**D)*S. capitis***

| **Level** | **- Level** | **Difference** | **Std Err Dif** | **Lower CL** | **Upper CL** | **p-Value** |
| --- | --- | --- | --- | --- | --- | --- |
| DDF_C | Healthy_O | 1.813 | 0.434 | 0.951 | 2.675 | <.0001* |
| DDF_C | Healthy_A | 1.505 | 0.442 | 0.629 | 2.382 | 0.0009* |
| DDF_O | Healthy_O | 1.122 | 0.434 | 0.260 | 1.984 | 0.0112* |
| DDF_O | Healthy_A | 0.814 | 0.442 | -0.063 | 1.691 | 0.0684 |
| DDF_C | DDF_O | 0.691 | 0.242 | 0.208 | 1.174 | 0.0056* |
| Healthy_A | Healthy_O | 0.308 | 0.267 | -0.225 | 0.840 | 0.2531 |

**E)*S. epidermidis***

| **Level** | **- Level** | **Difference** | **Std Err Dif** | **Lower CL** | **Upper CL** | **p-Value** |
| --- | --- | --- | --- | --- | --- | --- |
| Healthy_A | DDF_C | 1.209 | 0.300 | 0.615 | 1.804 | 0.0001* |
| Healthy_A | DDF_O | 0.900 | 0.300 | 0.306 | 1.494 | 0.0033* |
| Healthy_O | DDF_C | 0.847 | 0.294 | 0.264 | 1.429 | 0.0048* |
| Healthy_O | DDF_O | 0.537 | 0.294 | -0.045 | 1.120 | 0.0701 |
| Healthy_A | Healthy_O | 0.363 | 0.204 | -0.044 | 0.770 | 0.0800 |
| DDF_O | DDF_C | 0.309 | 0.186 | -0.060 | 0.679 | 0.0996 |
